# Randomized, Double-Blind, Placebo-Controlled, Single-Ascending-Dose Study of the Penetration of a Monoclonal Antibody Combination (ASN100) Targeting *Staphylococcus aureus* Cytotoxins in the Lung Epithelial Lining Fluid of Healthy Volunteers

**DOI:** 10.1128/AAC.00350-19

**Published:** 2019-07-25

**Authors:** Zoltan Magyarics, Fraser Leslie, Johann Bartko, Harald Rouha, Steven Luperchio, Christian Schörgenhofer, Michael Schwameis, Ulla Derhaschnig, Heimo Lagler, Leopold Stiebellehner, Christa Firbas, Susanne Weber, Ed Campanaro, Bernd Jilma, Eszter Nagy, Chris Stevens

**Affiliations:** a Arsanis Biosciences GmbH, Vienna, Austria; b Arsanis, Inc., Waltham, Massachusetts USA; c Department of Clinical Pharmacology, Medical University of Vienna, Vienna, Austria; d Department of Emergency Medicine, Medical University of Vienna, Vienna, Austria; e Department of Internal Medicine I, Division of Infectious Diseases and Tropical Medicine, Medical University of Vienna, Vienna, Austria; f Department of Internal Medicine II, Division of Pulmonology, Medical University of Vienna, Vienna, Austria; g EveliQure Biotechnologies GmbH, Vienna, Austria

**Keywords:** ASN100, phase 1, *Staphylococcus aureus* cytotoxins, anti-infective monoclonal antibodies, epithelial lining fluid pharmacokinetics, first-in-human trial

## Abstract

ASN100 is a novel antibody combination of two fully human IgG1(κ) monoclonal antibodies (MAbs), ASN-1 and ASN-2, which neutralize six Staphylococcus aureus cytotoxins, alpha-hemolysin (Hla) and five bicomponent leukocidins. We assessed the safety, tolerability, and serum and lung pharmacokinetics of ASN100 in a randomized, double-blind, placebo-controlled single-dose-escalation first-in-human study. Fifty-two healthy volunteers were enrolled and randomized to receive either ASN-1, ASN-2, a combination of both MAbs (ASN100), or a corresponding placebo. Thirty-two subjects in the double-blind dose escalation portion of the study received ASN-1 or ASN-2 at a 200-, 600-, 1,800-, or 4,000-mg dose, or placebo. Eight subjects received both MAbs simultaneously in a 1:1 ratio (ASN100) at 3,600 or 8,000 mg, or they received placebos. Twelve additional subjects received open-label ASN100 at 3,600 or 8,000 mg to assess the pharmacokinetics of ASN-1 and ASN-2 in epithelial lining fluid (ELF) by bronchoalveolar lavage fluid sampling. Subjects were monitored for 98 days (double-blind cohorts) or 30 days (open-label cohorts) for safety assessment. No dose-limiting toxicities were observed, and all adverse events were mild and transient, with only two adverse events considered possibly related to the investigational product. ASN100 exhibited linear serum pharmacokinetics with a half-life of approximately 3 weeks and showed detectable penetration into the ELF. No treatment-emergent anti-drug antibody responses were detected. The toxin neutralizing potency of ASN100 in human serum was confirmed up to 58 days postdosing. The favorable safety profile, ELF penetration, and maintained functional activity in serum supported the further clinical development of ASN100.

## Abstract (precis)

ASN100 is a novel antibody combination of two fully human IgG1(κ) monoclonal antibodies (MAbs), ASN-1 and ASN-2, which neutralize six Staphylococcus aureus cytotoxins, alpha-hemolysin (Hla) and five bicomponent leukocidins. We assessed the safety, tolerability, and serum and lung pharmacokinetics of ASN100 in a randomized, double-blind, placebo-controlled single-dose-escalation first-in-human study.

## INTRODUCTION

Monoclonal antibodies (MAbs) are established as safe and effective biologics for both the treatment and prevention of disease. To date, over 60 MAbs have received regulatory approval, including those used for both the treatment and the prevention of infectious diseases (1–3). Staphylococcus aureus is a human pathogen capable of causing infections ranging from mild conditions to severe diseases such as pneumonia and sepsis (4). S. aureus produces a multitude of cytotoxins that target epithelial cells and white blood cells (5, 6). Neutralization of S. aureus cytotoxins is viewed as a potential preemptive and therapeutic modality against staphylococcal infections (7).

ASN100 is a novel combination of two fully human IgG1(κ) MAbs, ASN-1 and ASN-2, that together neutralize six cytotoxins contributing to S. aureus pneumonia pathogenesis. ASN-1 neutralizes alpha-hemolysin (Hla or alpha toxin) and four bicomponent leukocidins: LukSF-PV (Panton-Valentine leukocidin), LukED, and two gamma-hemolysins HlgAB and HlgCB. ASN-2 neutralizes the fifth leukocidin, LukGH (also known as LukAB). Both MAbs demonstrated potent *in vitro* neutralizing activity in cell-based functional assays against target toxins (8, 9). They inhibit the assembly of pore complexes into target cell membranes but do not recognize toxin molecules after receptor binding (8–10). ASN-1 showed full protection in a lethal S. aureus pneumonia rabbit model (11), which was dependent on the neutralization of both Hla and bicomponent leukocidins (12, 13). In the same model, an Hla-only specific MAb protected only 25 to 33% of animals at a dose range of 10 to 30 mg/kg, while ASN100 afforded 100% survival against lethal challenge with a USA300 CA-MRSA at a 10 mg/kg dose (11, 13).

## RESULTS

### Investigational product administration and adverse events.

**FIG 1:**
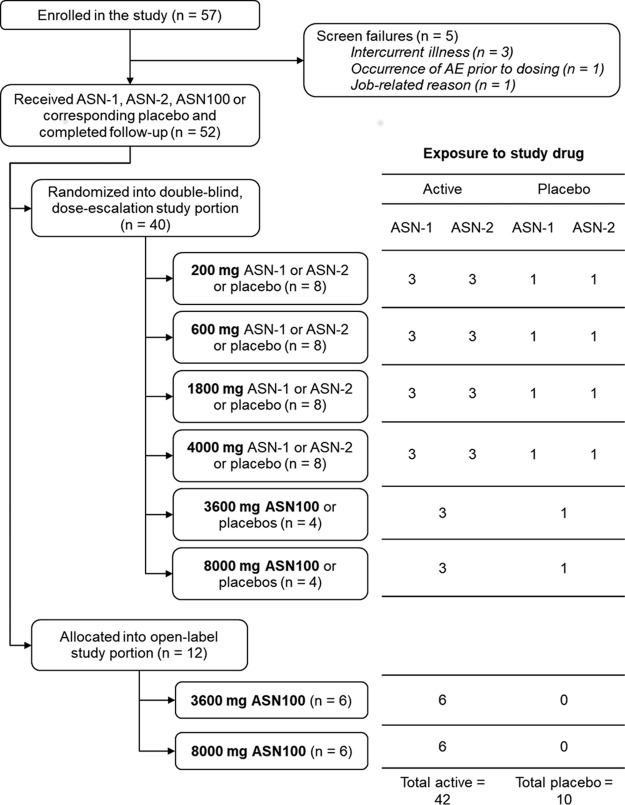
Flow chart of participant enrollment in the ASN100-01 trial outlines the disposition of subjects enrolled in the study, including screen failures and randomized subjects, as well as exposure to the study drug.

A total of 52 subjects were dosed between November 2015 and May 2016. Ten subjects received placebo, and 42 received ASN-1, ASN-2, or ASN100; exposure to the investigational product (IP) is summarized in Fig. 1. All 52 subjects attended all study visits and successfully completed the study. Subject demographics are summarized in Table 1 .

**TABLE 1:** Subject demographics and baseline characteristics^*a*^

Characteristic	Placebo, all doses (*n* = 10)	ASN-1 alone, all doses (*n* = 12)	ASN-2 alone, all doses (*n* = 12)	ASN100, double blind, all doses (*n* = 6)	ASN100, open label, all doses (*n* = 12)
Sex, no. (%)					
Male	6 (60.0)	9 (75.0)	9 (75.0)	6 (100.0)	11 (91.7)
Female	4 (40.0)	3 (25.0)	3 (25.0)	0	1 (8.3)
Race, no. (%)					
Caucasian	10 (100.0)	11 (91.7)	11 (91.7)	6 (100.0)	11 (91.7)
African-American	0	0	1 (8.3)	0	0
Asian	0	1 (8.3)	0	0	1 (8.3)
Mean age, yr (SD)	29 (6.36)	30 (8.04)	35 (10.17)	30 (5.35)	29 (9.73)
Mean body wt, kg (SD)	78 (12.8)	78 (11.4)	76 (10.2)	80 (12.1)	74 (8.7)
Mean ht, cm (SD)	179 (10.5)	178 (7.8)	179 (8.5)	181 (7.0)	181 (10.9)
Mean BMI, kg/m² (SD)	24.3 (3.4)	24.5 (3.0)	24.8 (3.1)	24.3 (2.7)	22.7 (2.0)

a *n*, number of subjects; BMI, body mass index. All doses, all dose levels.

ASN100 and its individual components were safe and well tolerated at doses up to 8,000 mg of ASN100 or 4,000 mg of its individual components, with no dose-limiting toxicities observed during the study; therefore, a formal maximum tolerated dose was not established. There were no related treatment-emergent abnormalities in any laboratory measure, and no clinically significant ECG changes were detected. The treatment-emergent adverse events (TEAEs) reported during the study are summarized in Table 2. The most frequently reported TEAEs were headache, nasopharyngitis, and symptoms of gastrointestinal infections (diarrhea, nausea, and abdominal pain). The TEAEs were balanced between ASN-1, ASN-2, ASN100, and placebo groups, with the exception of nervous system disorders. Only one subject reported headache among all subjects receiving placebo (10%) and pooled ASN-1 group (8.3%), while headache was observed in three subjects in the pooled ASN-2 group (25%) and seven subjects in the placebo-controlled and open-label pooled ASN100 groups (39%). Subjects receiving placebo did not report any other TEAEs classified as nervous system disorders, while three events were reported in subjects receiving active study drug: mild vertigo in a subject receiving 4,000 mg ASN-2, mild somnolence in a subject receiving 3,600 mg ASN100, and moderate dizziness in a subject receiving 8,000 mg ASN100. These events resolved without treatment and were deemed not related by the investigator. The frequency of TEAEs did not increase with higher doses. One unrelated SAE was reported in a subject who required surgical repair of a broken metatarsal bone due to an accident. All other TEAEs were transient, mild, or moderate in severity and resolved without intervention.

**TABLE 2:** Treatment emergent adverse events in subjects receiving ASN-1, ASN-2, ASN100, or placebo^*a*^

TEAE result	Placebo (all doses, *n* = 10)	ASN-1 or ASN-2			ASN100 (3,600 mg, *n* = 9*)	ASN-1 or ASN-2 (4,000 mg, *n* = 6)	ASN100 (8,000 mg, *n* = 9*)	ASN-1 alone (all doses, *n* = 12)	ASN-2 alone (all doses, *n* = 12)	ASN100 (all doses, *n* = 18†)
		200 mg, *n* = 6	600 mg, *n* = 6	1,800 mg, *n* = 6						
No. (%) of subjects reporting ≥1 TEAE	9 (90.0)	5 (83.3)	5 (83.3)	4 (66.7)	8 (88.9)	5 (83.3)	7 (77.8)	8 (66.7)	11 (91.7)	15 (83.3)
No. (%) of TEAE in system organ class										
Ear and labyrinth disorders	1 (10.0)	0	0	0	0	0	0	0	0	0
Gastrointestinal disorders	2 (20.0)	1 (16.7)	0	1 (16.7)	0	2 (33.3)	1 (11.1)	3 (25.0)	1 (8.3)	1 (5.56)
General disorders and administration site conditions	1 (10.0)	0	0	1 (16.7)	3 (33.3)	0	3 (33.3)	1 (8.3)	0	6 (33.3)
Infections and infestations	3 (30.0)	3 (50.0)	3 (50.0)	1 (16.7)	2 (22.2)	1 (16.7)	0	3 (25.0)	5 (41.7)	2 (11.1)
Investigations	3 (30.0)	0	0	0	0	0	1 (11.1)	0	0	1 (5.56)
Musculoskeletal and connective tissue disorders	2 (20.0)	0	2 (33.3)	1 (16.7)	1 (11.1)	2 (33.3)	0	2 (16.7)	3 (25.0)	1 (5.56)
Nervous system disorders	1 (10.0)	2 (33.3)	1 (16.7)	1 (16.7)	5 (55.6)	1 (16.7)	3 (33.3)	1 (8.3)	4 (33.3)	8 (44.4)
Psychiatric disorders	2 (20.0)	0	1 (16.7)	0	0	0	1 (11.1)	1 (8.3)	0	1 (5.56)
Renal and urinary disorders	1 (10.0)	0	0	0	0	0	0	0	0	0
Reproductive system and breast disorders	0	0	0	0	0	1 (16.7)	0	0	1 (8.3)	0
Respiratory, thoracic and mediastinal disorders	1 (10.0)	0	0	1 (16.7)	2 (22.2)	0	0	0	1 (8.3)	2 (11.1)
Surgical and medical procedures	0	0	1 (16.7)	0	0	0	0	0	1 (8.3)	0
Vascular disorders	1 (10.0)	0	0	0	1 (11.1)	0	1 (11.1)	0	0	2 (11.1)

a *n*, number of subjects. All doses, all dose levels. *, includes 6 subjects from open-label study phase with 30/37 days follow-up; †, includes 12 subjects from open-label study phase with 30/37 days follow-up.

Two subjects had TEAEs that were considered to be possibly related to the IP by the investigator. One of these was an episode of headache lasting for 12 h and reported to be mild in severity that occurred in a subject who received 200 mg ASN-1. The other was fatigue lasting for 12 days (between days 2 and 14 postdosing) and reported to be mild in severity that occurred in a subject who received 8,000 mg ASN100. Both events completely resolved without sequelae. No local infusion site reactions, systemic anaphylactic, or anaphylactoid reactions were reported. There were no deaths or IP discontinuations.

### Pharmacokinetics of ASN-1 and ASN-2.

**FIG 2:**
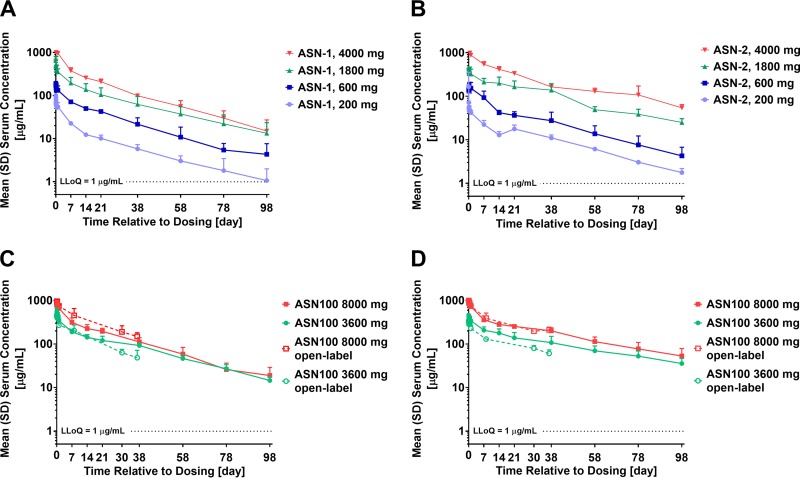
ASN-1 and ASN-2 pharmacokinetics in the serum of healthy adult volunteers after separate and simultaneous administration. (A and B) Mean serum ASN-1 (A) and ASN-2 (B) concentrations versus time up to 98 days after i.v. administration of ASN-1 or ASN-2 alone at 200-, 600-, 1,800-, or 4,000-mg doses (three subjects/dose/MAb). (C and D) Mean serum ASN-1 (C) and ASN-2 (D) concentrations versus time up to 98 days (double-blind cohorts; filled symbols) or up to 37 days (open-label cohorts; open symbols) after simultaneous i.v. administration of ASN-1 and ASN-2 each administered as 1,800- or 4,000-mg doses (in combination designated as 3,600 mg ASN100 or 8,000 mg ASN100, respectively). Error bars indicate ± the standard deviations (SD). Horizontal lines indicate the lower limit of quantification (LLoQ) at 1 μg/ml.

ASN-1 and ASN-2 exhibited linear serum PK with limited intersubject variability both when administered alone (Fig. 2A and B) or in combination (Fig. 2C and D). Serum exposure, characterized by *C*_max_, AUC_0–d98_, and AUC_0–∞_, increased approximately dose proportionally over the full range of doses tested (Table 3). Clearance (CL) and the volume of distribution at steady state (*V*_ss_) were comparable across all dose levels (Table 3).

**TABLE 3:** ASN-1 and ASN-2 serum pharmacokinetic parameters for each dose by noncompartmental analysis^*a*^

IP and dose (no. of subjects)	Mean (%CV)											
	*C*_max_ (μg/ml)		AUC_0–d98_ (μg ⋅ h/ml)		AUC_0–∞_ (μg ⋅ h/ml)		Clearance (liters/h)		*V*_ss_ (liters)		Half-life (days)	
	ASN-1	ASN-2	ASN-1	ASN-2	ASN-1	ASN-2	ASN-1	ASN-2	ASN-1	ASN-2	ASN-1	ASN-2
ASN-1 or ASN-2												
200 mg (*n* = 6)	100 (10.3)	165 (114)	17,948 (22.0)	24,242 (17.9)	19,411 (21.7)	25,751 (17.6)	0.0107 (24.8)	0.00793 (17.9)	6.89 (3.1)	6.58 (24.4)	25.3 (28.4)	23.9 (0.7)
600 mg (*n* = 6)	185 (13.5)	183 (18.7)	60,515 (13.5)	67,100 (34.6)	64,516 (16.3)	70,440 (35.5)	0.0095 (15.3)	0.00936 (38.9)	6.63 (18.8)	6.10 (21.6)	25.0 (32.4)	31.3 (16.6)
1,800 mg (*n* = 6)	578 (41.3)	453 (4.9)	172,066 (38.9)	244,728 (19.4)	183,930 (41.6)	269,507 (20.1)	0.0108 (33.5)	0.00686 (19.4)	8.02 (32.3)	6.38 (12.3)	24.0 (20.1)	28.3 (10.9)
4,000 mg (*n* = 6)	1,868 (3.5)	1,342 (5.5)	339,474 (15.6)	530,058 (10.1)	351,387 (15.1)	593,307 (9.6)	0.0116 .(154)	0.00678 (9.6)	7.00 (32.2)	6.78 (13.7)	19.7 (31.9)	32.8 (23.1)
												
ASN100												
3,600 mg (*n* = 3)	550 (20.6)	468 (0.7)	201,134 (132.2)	250,887 (21.9)	213,265 (14.1)	296,315 (21.7)	0.0086 (15.0)	0.00627 (21.6)	6.89 (7.5)	7.55 (21.6)	24.1 (4.3)	36.3 (17.4)
3,600 mg (*n* = 6), open label	638 (25.1)	372 (11.8)	NA	NA	135,631 (15.8)	151,232 (13.8)	0.0136 (18.5)	0.01207 (11.8)	6.37 (33.8)	9.4 (12.7)	14.0 (26.2)	22.8 (23.5)
8,000 mg (*n* = 3)	975 (12.7)	1,075 (12.7)	302,392 (16.7)	437,901 (13.2)	316,427 (18.6)	503,209 (20.1)	0.0130 (18.3)	0.00816 (19.5)	8.24 (7.6)	8.26 (10.8)	20.3 (14.9)	32.3 (31.3)
8,000 mg (*n* = 6), open label	1,252 (33.0)	1,095 (16.5)	NA	NA	342,449 (29.2)	382,996 (19.5)	0.0124 (23.5)	0.01078 (19.4)	5.54 (18.6)	6.93 (20.1)	13.2 (13.2)	18.3 (9.1)

a Data are expressed as means (percent coefficients of variation [%CV]). IP, investigational product; NA, not applicable.

The terminal elimination half-life (*t*_1/2_) ranged from 472 to 608 h (20 to 25 days) for ASN-1 and from 520 to 870 h (22 to 36 days) for ASN-2 across dose levels in the double-blind study portion. Values were similar between corresponding dose levels when ASN-1 or ASN-2 were infused alone or coadministered, with one exception when administered at the 1,800-mg dose level. At this dose level, the *t*_1/2_ was 1.3-fold higher when ASN-2 was coadministered with ASN-1 compared with ASN-2 alone, but all other PK characteristics were comparable between ASN-2 alone versus together with ASN-1.

### Epithelial lining fluid pharmacokinetics of ASN-1 and ASN-2.

**FIG 3:**
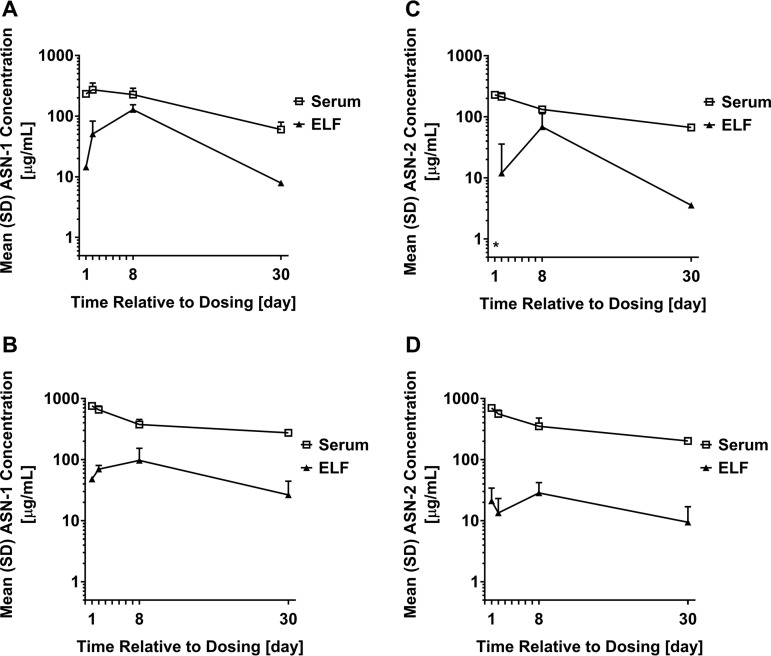
ASN-1 and ASN-2 pharmacokinetics in the serum and epithelial lining fluid of healthy adult volunteers. Mean serum (rectangles) and ELF (filled triangle) concentrations are shown of ASN-1 (A and B) and ASN-2 (C and D) versus time up to 30 days after simultaneous i.v. administration of ASN-1 and ASN-2, each administered as 1,800-mg (A and C) or 4,000-mg (B and D) doses. Error bars indicate the SD; *, below the limit of antibody quantification.

Measurable levels of ASN-1 and ASN-2 were detected in epithelial lining fluid (ELF) at both doses tested as early as day 1 and out to day 30 postdosing, with significant intersubject and intrasubject variability over time (Fig. 3). The highest ASN-1 and ASN-2 concentrations were observed on day 8 postdose. ELF PK parameters are summarized in Table 4.

**TABLE 4:** ASN100 Epithelial lining fluid pharmacokinetic parameters for each dose determined by noncompartmental analysis

Analyte	Treatment (dose, mg)	Mean AUC_0–_*_t_* (μg ⋅ h/ml)^*a*^	*C*_max_ (μg/ml)		*T*_max_ (h)
			Mean (SD)	Median	
ASN-1	ASN100 (3,600)	50,993 (5,331.1)	128 (25.8)	128	192
	ASN100 (8,000)	46,632 (9,637.9)	97 (56.0)	89	192
ASN-2	ASN100 (3,600)	24,498 (8,331.6)	69 (43.8)	55	192
	ASN100 (8,000)	13,702 (2,729.2)	28 (13.5)	27	192

a Standard errors are indicated in parentheses.

### Neutralizing activity of ASN100 in sera of study subjects.

**FIG 4:**
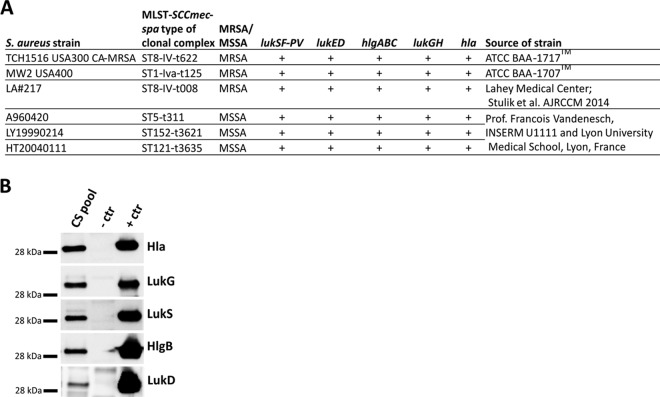
S. aureus strains used in this study. (A) Genetic characterization and source. (B) Immunoblot analysis of the pooled CS of the six S. aureus strains listed in panel A. A 0.1-μg portion of recombinant toxin was used as a positive control (+ctr); a culture supernatant generated with an isogenic mutant of TCH1516 strain lacking the genes for Hla and all five bicomponent toxins was included as a negative control (–ctr).

**FIG 5:**
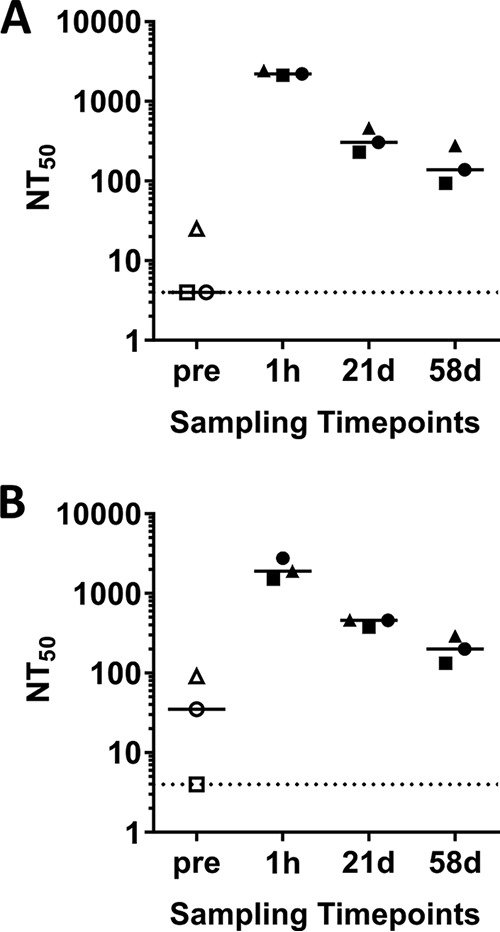
Neutralization of native S. aureus toxins with serum samples obtained from study subjects treated with ASN100. Serially diluted pre- and postdosing serum samples of study subjects receiving 3,600 mg of ASN100 in the double-blind portion of the study were analyzed for neutralization of Hla toxicity toward human lung epithelial cells (A) or neutralization of leukocidin toxicity toward human neutrophils (B) induced by toxins in S. aureus culture supernatant. The neutralization potency was measured in ATP-based cell viability assays and is expressed as the NT_50_ titer. Group medians are indicated by horizontal lines; different symbols represent the individual subjects. The values shown were calculated based on the results of two independent experiments. Dashed lines indicate the cutoff value in functional toxin inhibition assays.

**FIG 6:**
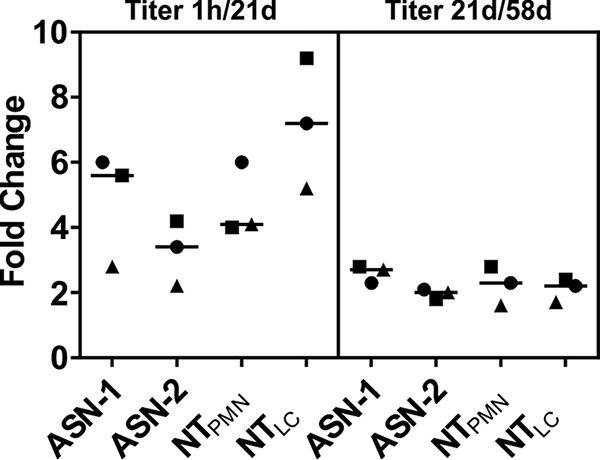
Kinetics of changes in cytotoxin neutralization titers and MAb serum levels. Fold changes in serum MAb levels and PMN (leukocidin) and lung cell (LC; Hla) toxicity neutralization titers postdosing at day 21 relative to 1 h and at day 58 relative to day 21 are depicted. Group medians are indicated by horizontal lines; different symbols represent individual subjects.

The functionality of ASN100 in human serum and its additive effects in the presence of preexisting S. aureus toxin neutralizing antibodies was analyzed in two types of *in vitro* assays, using either human lung epithelial cells or human neutrophils (PMNs) as target cells for Hla and the bicomponent leukocidins, respectively. Six different S. aureus strains (see Fig. 4A) were cultured, and a mixture of their early-stationary-phase culture supernatants with confirmed expression of Hla and the five bicomponent leukocidins (Fig. 4B) was used in the assays. The predosing sera of the three subjects in the 3,600-mg ASN100 double-blind dose group displayed variable Hla and leukocidin neutralization titers (NT_50_) between <4 to 25 and <4 to 90, respectively. The postdosing samples had comparable and at least 5-fold increased toxin neutralization potencies at each sampling time point (Fig. 5). The NT_50_ values on day 21 posttreatment were up to 2-log higher than values in the predosing sera of subjects with initially low neutralization titers (≤4). The kinetics of leukocidin and Hla neutralization titers reflected changes in ASN-1 and ASN-2 serum levels, confirming that functional activity (antigen binding) of the MAbs was not altered (Fig. 6).

### Immunogenicity of ASN100: anti-drug antibodies.

Serum samples of all subjects in the double-blind cohorts were tested for the presence of anti-ASN-1 and anti-ASN-2 anti-drug antibodies (ADAs). No subjects developed new onset, treatment-emergent ADAs. Two subjects tested positive for anti-ASN-1 ADA before administration of the IP (baseline), and one of these subjects tested positive at day 98 after administration of 600 mg ASN-1. There was no increase in ADA titers after IP administration, and the presence of preexisting ADA was not associated with any adverse events (AEs) or any impact on ASN100 PK in the two subjects who tested positive for anti-ASN-1 ADA at baseline. None of the subjects tested positive for anti-ASN-2 ADA.

## DISCUSSION

The aim of this first-in-human, phase 1, single-ascending-dose study of ASN100 and its components (ASN-1 and ASN-2) was to evaluate the safety, tolerability, and PK of the MAbs alone and in combination in healthy volunteers.

The administration of ASN100, as well as its individual components administered separately, did not result in any treatment related SAEs or study discontinuations. There was no dose-limiting toxicity up to 8,000 mg ASN100. The treatment-emergent AE profile was balanced between active and placebo groups, with the exception of nervous system disorders, where subjects receiving active investigational medicinal product reported more events of headache (26.2% of subjects in active groups versus 10% of subjects in placebo groups) and single events of somnolence, vertigo, and dizziness. There were no AEs associated with dose escalation of ASN-1, ASN-2, or ASN100 and only two mild AEs of headache and fatigue that were assessed as possibly treatment related. Lastly, there were no AEs suggestive of ECG abnormalities of hypersensitivity reactions. These findings are consistent with those of other fully human MAbs, where slight imbalance in frequency of headache and other nervous system TEAEs was also observed (3, 14–17).

Both ASN-1 and ASN-2 exhibited approximately linear PK with an observed half-life of 20 to 36 days at doses up to 4,000 mg administered as a single intravenous (i.v.) dose alone and in combination in the double-blind study portion, consistent with the PK of other human IgG1 antibodies (18, 19). The serum PK of ASN-1 and ASN-2 administered simultaneously was comparable to the PK of the MAbs given alone, suggesting no clinically significant PK interaction between the two MAbs. ASN-1 exposure (AUCs) was comparable when ASN-1 was infused alone or in combination with ASN-2 (ASN100). For ASN-2, a somewhat longer *t*_1/2_ was observed when administered with ASN-1 compared to administration alone, but no differences were observed in the rate and extent of ASN-2 exposure, CL, or *V*_ss_. In general, a dose proportional increase in peak concentration and exposure was observed across all dose levels of ASN-1 or ASN-2 alone. When ASN-1 and ASN-2 were coadministered (ASN100), the ASN-1 total (AUC_0–∞_) and the peak exposure increased in a slightly less than dose-proportional manner, while ASN-2 total exposure increased in a dose-proportional manner. Other than this exception, the overall serum PK of ASN-1 and ASN-2 were comparable when given alone or together.

To support clinical indications of S. aureus pulmonary infections, ASN100 PK were assessed in ELF by measuring the concentrations of ASN-1 and ASN-2 via bronchoalveolar lavage (BAL) fluid sampling in healthy volunteers. After a single i.v. infusion, ASN-1 and ASN-2 were detectable in the first samples taken within 24 or 48 h and remained detectable in ELF at least out to day 30 postdose. The time point of peak ASN-1 and ASN-2 concentration in ELF could not be fully determined and might fall between day 2 and 8 or after day 8. No clear dose response was observed in the ELF concentrations at the two doses tested. However, to our knowledge the penetration of potentially therapeutic MAbs into the human lung has not been reported. ELF concentrations of human MAbs were previously measured in relevant animal models (20, 21).

Limitations of these analyses were the small sample size of each cohort in the dose-escalation portion and the shorter follow-up period and limited number of BAL samplings (*n* = 2 at each time point) in the open-label portion, making the pharmacokinetic (PK) parameter estimates less accurate. Future studies will explore the serum and lung PK of ASN100 in target patient populations.

To assess the toxin neutralizing potency of ASN100 present in the sera of subjects dosed with ASN100, pre- and postdosing serum samples of the cohort receiving 3,600 mg ASN100 were analyzed. Sera containing ASN100 MAbs had significantly increased toxin neutralization titers up to 58 days postdosing compared to the corresponding predosing samples (Fig. 5). The comparable kinetics between serum ASN-1 and ASN-2 levels and neutralization titers confirmed that the functional activity of the MAbs were retained in human sera out to day 58 postdosing.

It is well documented that the efficacy and safety of therapeutically administered MAbs can be altered by ADAs generated in treated subjects. We did not detect any treatment-emergent ADAs against ASN-1 and ASN-2 after single-dose administration, although the free drug tolerance of the ADA assays limited the ability to detect ADAs in higher dose cohorts and at early time points. The two subjects with confirmed ADA positivity against ASN-1 had detectable preexisting antibody levels that did not increase upon ASN-1 exposure. The preexisting ADAs were not associated with any impact on ASN100 safety or serum PK.

### Conclusions.

This first-in-human trial demonstrated that ASN100 was safe and well tolerated in healthy, adult volunteers at doses up to 8,000 mg. In addition ASN100 pharmacokinetics were predictable, approximately linear, and dose dependent for all dose levels tested, and the functional activity of MAbs from human sera was confirmed. Both ASN-1 and ASN-2 were detected in ELF at early time points and at concentrations anticipated to achieve clinical effect in patients and sustained concentrations of both MAbs in the ELF out to day 30 postdose. These observations in conjunction with the safety and tolerability profile supported the continued clinical development of ASN100 in a randomized controlled phase 2 trial for the prevention of S. aureus pneumonia in mechanically ventilated patients.

## MATERIALS AND METHODS

### Study objectives and design.

This study was conducted at the Department of Clinical Pharmacology at the Medical University of Vienna, Austria.

In the double-blind, placebo-controlled dose escalation phase, 40 healthy volunteers were randomized to one of ten fixed-dose cohorts, in a 3:1 ratio to receive either active treatment or placebo(s), infused over 50 to 60 min. Active treatment was ASN-1 or ASN-2 administered alone (doses of 200, 600, 1,800, and 4,000 mg) or in combination as ASN100 (dose levels of 3,600 and 8,000 mg; administered as a 1:1 ratio of ASN-1 and ASN-2 simultaneously via separate i.v. lines). The primary endpoint was the safety and tolerability of a single dose of ASN100 and its individual antibody components (ASN-1 and ASN-2). The secondary objectives were to evaluate the PK of ASN100 and its individual MAb components, and to measure the ADA levels in serum against both MAbs.

Subjects and all study personnel were blinded, with the exception of the clinical pharmacist who prepared the investigational product (IP). The study duration included a screening period of up to 14 days prior to IP administration and a follow-up period of 98 days postdosing. Subjects were dosed at least 24 h apart between two-subject groups and monitored for 7 days postdose prior to dose escalation. Dose escalation was approved by an independent Data and Safety Monitoring Board (DSMB) following review of blinded safety data. Further, after completion of IP administration in the ASN-1 and ASN-2 single MAb cohorts, administration of ASN100 was approved by the DSMB, the local independent ethics committee (IEC), and the Austrian National Competent Authority BASG/AGES.

After completion of the double-blind study portion, 12 additional subjects in two open-label cohorts (6 subjects each) received 3,600 or 8,000 mg of ASN100. These cohorts were included to measure concentrations of ASN-1 and ASN-2 in the epithelial lining fluid (ELF) at two time points by BAL fluid sampling within 30 postdosing. The study duration for these cohorts included a screening period of up to 14 days prior to IP administration and a follow-up period of 30 days except for those subjects undergoing BAL fluid collection on day 30, where it was 37 days. Since no statistical hypothesis was tested, a formal sample size calculation was not performed.

Participant flow and exposure to ASN-1, ASN-2, ASN100, and corresponding placebo(s) are summarized in Fig. 1.

### Inclusion and exclusion criteria.

Volunteers between 18 and 55 years of age, healthy based on medical history and physical examination at screening, weighing between 60 and 100 kg and with a body mass index (BMI) of <30 kg/m^2^, were eligible to participate. Subjects had to meet criteria for normal baseline physical examination and laboratory assessment, including electrocardiogram and negative screening assessments (or recent screening results) for human immunodeficiency virus and viral hepatitis (hepatitis B or hepatitis C). Subjects with a history of anaphylaxis or documented severe hypersensitivity reaction and/or injection site reaction, with current or prior use of immunoglobulin products, or receiving current or recent (<1 month) immunosuppressive therapy with steroids, immunomodulators, or anti-inflammatory drugs other than nonsteroidal anti-inflammatory drugs were excluded. Written informed consent (approved by the IEC) was obtained from subjects before study procedures began. The study was conducted in accordance with the ethical principles of the Declaration of Helsinki and the International Council for Harmonization Guidelines on Good Clinical Practice, as well as Austrian local regulations. The study was registered in the EudraCT database (2015-003144-39).

### Investigational product and placebo.

ASN-1 and ASN-2 were produced by Boehringer Ingelheim Biopharmaceuticals GmbH (Ingelheim, Germany) in stably transfected CHO cells according to Good Manufacturing Practice standards and were formulated at a 20-mg/ml concentration in proprietary formulations. The formulation buffers were used as placebos.

### Assessments.

Vital signs were frequently assessed from initiation of the investigational product infusion until 24 h after completion of administration. Safety assessments included recording of AEs occurring from initiation of IP administration dosing through 98 days postdose (30 or 37 days postdose for the open-label cohorts).

To assess the serum PK profile and the presence of ADA, serum samples were collected prior to IP administration on day 1, immediately after dosing, hourly until 6 h, and then at 12 and 24 h and on days 7, 14, 21, 38, 58, and 98 postdose. For the open-label cohorts, additional samples were collected concurrently with BAL fluid collection, as well as, on days 8 and 30 or 37 postdose. The presence of ADA was assessed at predosing and on day 14, 38, and 98 postdose (day 30 or 37 for open-label cohorts).

### Collection of bronchoalveolar lavage fluid samples.

For subjects enrolled in the open-label study cohorts, bronchoscopic sampling was performed to obtain BAL fluid either on days 1 and 30 or on days 2 and 8, according to the current guidelines (22, 23) and as described previously (24). During bronchoscopy, a total of 100 to 300 ml of prewarmed sterile isotonic saline was instilled and retrieved in three to five aliquots through the bronchoscope from the middle lobe or the lingula. BAL fluid was collected on ice and immediately centrifuged for 10 min at 900 × *g* at 4°C, and the supernatant was stored at –80°C.

### Pharmacokinetic analysis of ASN-1 and ASN-2.

Concentrations of ASN-1 and ASN-2 in the serum and the BAL fluid were measured using an enzyme-linked immunosorbent assay (ELISA). Immobilized anti-ASN-1 or anti-ASN-2 anti-idiotype antibodies (custom produced by AbD Serotec/Bio-Rad, Puchheim, Germany) were used to capture ASN-1 and ASN-2 in human serum samples or BAL fluid and horseradish peroxidase-conjugated goat anti-human Fc polyclonal antibody (Jackson Immuno Research, West Grove, PA) was used for detection. ASN-1 and ASN-2 concentrations were determined by interpolating the observed endpoint value from a four-parameter logistic function, standard curve-fit generated using SoftMax Pro v6.2.2 software (Molecular Devices, San Jose, CA). Assay validation was performed with human serum as matrix. The lower limit of quantification (LLoQ) in undiluted serum samples was 1 μg/ml for both ASN-1 and ASN-2; the intraassay and interassay precision remained within the criteria (coefficient of variation [CV] ≤ 20% for the LLoQ control sample, CV ≤ 15% for all other quality control samples) defined in the relevant guidelines of the U.S. Food and Drug Administration and the European Medicines Agency (25, 26). Importantly, the validation of ASN-1- and ASN-2-specific ELISA methods included the selectivity assessment to exclude cross-detection of the nonassayed antibody component of ASN100.

Concentrations of ASN-1 and ASN-2 in BAL fluid were determined using the same methods as for serum PK, except that validation of the assays was not performed with BAL fluid as matrix. The LLoQ in undiluted BAL fluid were 0.04 μg/ml for ASN-1 and 0.2 μg/ml for ASN-2. BAL fluid concentrations of ASN-1 and ASN-2 were normalized to dilution-corrected ELF concentrations by urea-based normalization to compensate for the BAL fluid dilution, using serum urea (calculated from blood urea nitrogen) and BAL fluid urea concentrations (measured using a validated enzymatic assay [QuantiChrom Urea Assay kit; Bioassay Systems, Hayward, CA]) to estimate the dilution factor of the BAL fluid (27). Six ELF samples were excluded from the final analysis due to analytical reasons; four of these were excluded from ASN-1 PK analysis due to imperfect dilution linearity of the ASN-1 measurement, and two of them were completely excluded due to low precision (CV >25%) of the replicate measurements in the urea assay.

The pharmacokinetics (PK) of ASN-1 and ASN-2 were determined by noncompartmental methods using a validated version of Phoenix WinNonlin (v6.3). The *C*_max_ was defined as the maximum observed concentration. The terminal elimination half-life was determined using a log-linear regression of the concentration data with the equation ln_2_/λ_Z_, where the terminal elimination rate constant λ_Z_ is the slope of the terminal portion of the natural log concentration-time curve, calculated from at least three data points in the terminal phase of the concentration-time profile. The systemic ASN-1 and ASN-2 exposure was determined by calculating the area under the serum concentration-time curve (AUC) from time zero to the last quantifiable concentration (AUC_0–_*_t_*) or to infinity (AUC_0–∞_), using a linear-logarithmic trapezoidal method. Clearance (CL) was calculated as dose/AUC_0–∞_. The volume of distribution at steady state (*V*_ss_) at steady-state was calculated as MRT ⋅ CL, where MRT = (AUMC_0–∞_/AUC_0–∞_) – ID/2, where AUMC_0–∞_ is the area under the first moment curve extrapolated to infinity, and ID represents infusion duration.

### Detection of anti-ASN-1 and anti-ASN-2 serum antibodies.

Validated electrochemiluminescent, solution-phase, bridging immunoassays were used for detection and quantification of anti-ASN-1 and anti-ASN-2 antibodies in human serum. Biotinylated ASN-1 or ASN-2 antibodies were bound to streptavidin-coated mesoscale discovery microplates to capture ADAs present in serum samples. ADAs were detected using ruthenylated ASN-1 or ASN-2. The assay used anti-ASN-1 and anti-ASN-2 anti-idiotype antibodies (custom produced by AbD Serotec/Bio-Rad) as positive controls. Samples with electrochemiluminescent responses equal to or above the cut point value defined as floating cut point for each individual analytical run according to the methods of Shankar et al. (28) were considered positive in screening. Confirmation of ADA positivity was performed by spiking human serum samples with 10 μg/ml of ASN-1 or ASN-2 prior to the ADA assay. ADA positivity was confirmed if the percent inhibition of ADA binding by spiked-in ASN-1 or ASN-2 was above the confirmatory cut point (33.45% for anti-ASN-1 and 14.39% for anti-ASN-2 assessment). The sensitivities of ADA detection were 0.76 ng/ml for anti-ASN-1 and 1.56 μg/ml for anti-ASN-2 antibodies, and the free drug tolerances were 1.25 μg/ml for anti-ASN-1 and 1.46 μg/ml for anti-ASN-2 antibodies. Since the free drug levels exceeded the drug tolerance limit of the ADA assays in all postdose serum samples of subjects enrolled in the open-label cohorts due to the shorter follow-up, these samples were not tested for the presence of ADA.

### Determination of toxin neutralizing activity of ASN100 in human serum samples.

Toxin neutralizing activity of serum samples obtained from the three study subjects dosed with 3,600 mg ASN100 (double-blind cohort) was tested in cell-based assays. Alpha-hemolysin and leukocidin neutralization potencies of serum samples collected before and after ASN100 administration, obtained from the three study subjects dosed with 3,600 mg of ASN100 in the double-blind portion of the study, were assessed using a human alveolar epithelial cell line (A549, ATCC CCL-185) and human PMNs, respectively. PMNs were freshly isolated from heparinized human blood as described previously (8, 10). Serum samples collected before ASN100 administration and at three time points (1 h, 21 days, and 58 days) after administration were serially diluted in assay medium (RPMI 1640 supplemented with 10% fetal calf serum [FCS] and l-glutamine for the PMN assays and F12K medium supplemented with 10% FCS for A549 assays) and preincubated with S. aureus culture supernatant (CS), used as the source of native toxins, for 30 min. The CS used in this study was a pool generated from early stationary cultures of six S. aureus strains, all carrying *lukSF-PV* (see Fig. S3A in the supplemental material), grown in low-iron RPMI medium supplemented with 1% Casamino Acids. The CS pool was prepared as described before (9) and used at 6× and 12× final dilutions for Hla and leukocidin neutralization assays, respectively. The presence of ASN100-targeted cytotoxins in the CS pool was confirmed by immunoblot analysis (Fig. S3B) as described previously (9). For both cell types, cell viability was measured by quantification of cellular ATP content using a Cell Titer-Glo luminescent cell viability assay kit (Promega, Madison, WI) as described before (8, 9). The percent viability was calculated relative to untreated control cells. Data were analyzed using Prism 6 (GraphPad, La Jolla, CA). The toxin neutralizing activity of the sera was expressed as the 50% neutralizing titer (NT_50_), the reciprocal of the serum dilution that was required to inhibit cytotoxicity by 50%.
